# The role of duration and frequency of occurrence in perceived pitch structure

**DOI:** 10.1371/journal.pone.0239582

**Published:** 2020-09-21

**Authors:** Michael E. Lantz, Anja-Xiaoxing Cui, Lola L. Cuddy

**Affiliations:** 1 Department of Psychology, Queen’s University, Kingston, Ontario, Canada; 2 School of Music, University of British Columbia, Vancouver, British Columbia, Canada; University College London, UNITED KINGDOM

## Abstract

**Introduction:**

To survive, organisms need to organize perceptual input into coherent, usable structures. Research has illuminated the potential role of frequency of occurrence and duration as cues to extract statistical regularities from our environment. Musical stimuli provide a unique opportunity to study how these cues are used to organize auditory input into higher level perceptual entities, i.e., pitch structure, and to assess the influence of cognitive schema.

**Methods:**

To examine the relative importance of these two cues in pitch structure perception, we constructed novel tone sequences in which frequency of occurrence and duration cues were pitted against each other. We assessed perceived pitch structure in musically trained and untrained listeners using a probe tone paradigm.

**Results:**

In all experiments, a 3-tiered hierarchy of pitch structure emerged, with highest ratings for tones of longer duration, next highest for shorter, more frequent tones and lowest for probe tones that did not occur in the sequence. The hierarchy did not reflect assimilation to Western tonal schema.

**Discussion:**

Our results argue against theories positing the same mechanism for the processing of duration and frequency of occurrence, and that duration is weighted preferentially. We further suggest that the organization of perceptual information will proceed according to whatever information is relevant, available, and most easily acquired.

## Introduction

Humans, and other organisms, actively extract regularities from the environment in order to organize the information into coherent, useable structures [1]. Sensitivity to statistical regularities is essential to abstracting meaning from the environment as well as to tracking ongoing changes in the environment [2]. Such sensitivity can be shown in infants as young as 8-months old [3–6]. Through long-term exposure, statistical regularities may be internalized as schemas, providing a framework for the organization and processing of ongoing and novel events [7, 8]. Thus, studying what kind of statistical regularities may be used to form schemas is crucial to a comprehensive understanding of how we make sense of our environment.

### Two regularities to which we are sensitive—Frequency of occurrence and duration of events

Frequency of occurrence and duration are two primary attributes of all events in all modalities. Every event must occur and each occurrence must have some duration. Many studies have been conducted on the behavioral consequences and the neural substrates responsible for processing these cues. We will now present in turn what we perceive to be the most relevant evidence for our sensitivity to frequency of occurrence and duration and their function in perception.

#### Frequency of occurrence

People are extremely accurate at estimating the frequency of occurrence of events within lists of items such as words or pictures [9–16] although accuracy is over- or underestimated in certain circumstances [17–20]. The ability to accurately estimate frequency of occurrence information develops early in life [21, 22] and, for visual items, is processed early in the visual pathway [23]. Thus, it can be considered an important cue that we use to make sense of our environment.

Frequency of occurrence information is not only readily available but it is useful across a range of different types of tasks. Studies have highlighted the effect of increasing frequency of occurrence on word learning in infants and individuals with aphasia [24, 25]. Increased frequency of occurrence also supports pitch recognition [26]. Furthermore, children who spontaneously focus on frequency of occurrence information tend to have better arithmetic skill [27–29].

#### Duration

In a similar fashion, many studies have shown the importance of duration of events in perception and cognition. For example, longer exposure times lead to better face recognition [30], better photograph recognition [31, 32], higher confidence ratings for complex picture recognition [33], and better word recognition [34]. Duration is also a very salient feature of events around us. Infants as young as 8 months old, for example, are sensitive to duration and can use it to organize the structure of events [6]. Stress patterns in speech created at least in part by duration lead to the segmentation of words from speech streams, even by infants [35–37]. In music, duration can create temporal patterns that create accents on the longer tones, making those tones more salient [38, 39].

### One or two cues?

There is a fundamental difference between frequency of occurrence and duration. Increments in frequency of occurrence information happen in discrete steps whereas increments in duration are continuous [40]. However, a long-standing debate revolves around whether frequency of occurrence information and duration information are encoded as the same cue or separate cues [10–12, 14]. Some researchers [41–43] have suggested an information processing approach in which both duration and frequency of occurrence act on a single mechanism that gates into a timing or counting switch. According to these authors, tasks requiring timing or counting rely on the single mechanism. Walsh [44–46] also argued for a single accumulator that encodes any kind of magnitude information, including frequency of occurrence, duration, size, and space. Walsh’s theory rests on evidence from several lines of research.

First, the psychophysical functions for both cues are similar in rodents as well as in humans [42, 43, 47, 48]. Second, neural imaging techniques show that the pathways for both frequency of occurrence and duration involve overlapping areas of the parietal cortex [49–57]. Third, damage to such parietal areas leads to the overlap of deficits in time, number, space, and size processing [58]. Fourth, processing of one type of magnitude information interferes with other types of magnitude information [59].

However, Walsh’s [44–46] first three points are not necessarily evidence in favor of a single mechanism over a multiple mechanism system. For example, the fact that psychophysical functions for both cues are similar does not necessitate a sharing of neural circuits. Separate circuits could be processing information in similar ways. Further, Droit-Volet et al. [47] showed that the discrimination function in choosing between two durations is different than the discrimination function in choosing between two frequencies of occurrence showing that not all psychophysical functions for both cues are similar. Roitman et al. [48] found similar functions in frequency-of-occurrence and duration information but found that participants were less sensitive to duration information suggesting a separation of the two cues.

Neural overlap does not necessarily mean a sharing of neural circuits [60, 61]. Even with overlapping activation, only a portion of neurons in a given region could be responding to a given process [62]. Pesenti, Thioux, Dormal, De Volder, and Seron [63] specifically sought and failed to find overlapping parietal activation for frequency of occurrence and duration tasks. And Dormal et al. [50] found that duration processing but not frequency of occurrence processing was impaired in elderly Parkinson’s Disease patients compared to elderly and younger controls. Each of these findings suggests that the two cues operate through independent mechanisms.

Contributing to this debate are studies examining the interference of these cues. If both cues interfere with each other, it could be argued that there is a single mechanism for both. A one-sided interference or the absence of interference would argue for more than one mechanism. Results of these interference studies have been equivocal. On the one hand, Javadi and Aichelburg [59] found interference by covarying the frequency of occurrence of items in a visual display and the duration with which the items were displayed. Frequency of occurrence estimates increased as the items in the display increased, and estimates of duration increased with increasing frequency of occurrence. Each cue was influenced by the other, so they may not be considered independent. On the other hand, other researchers [64–66] found one-sided interference. They asked participants to estimate either the number of times a dot flashed or the total duration of the flashing. Frequency of occurrence interfered with duration estimates whereas duration did not interfere with frequency of occurrence estimates. And different still, Agrillo, Ranpura, and Butterworth [67] asked participants to either judge the duration of a tone sequence overall, or how many tones were in the tone sequence and found no interference effects.

There is further evidence in favor of separation of the cues. Dormal, Javadi, Pesenti, Walsh, and Cappelletti [68] used transcranial random noise stimulation on parts of the right parietal cortex and found enhanced duration processing but no change in frequency of occurrence categorization. Such a differential effect is evidence against the idea of a single magnitude accumulator. Another aspect to consider is whether there are differences between the functional use of the frequency of occurrence and duration cues in a behavioral task. Breukelaar and Dalrymple-Alford [69] taught rats to discriminate between few versus many sound bursts and also taught the same rats to discriminate between short versus long sound bursts. The rats learned to discriminate durations faster and better than they learned to discriminate frequencies of occurrence. Moreover, when later tested on differentiation tasks in which each cue required a different response, rats responded to duration cues while ignoring frequency of occurrence cues. If frequency of occurrence and duration are functionally the same cue, the rats should not have systematically responded to one cue over the other.

Although there is evidence for a single mechanism for magnitude, some of the evidence is ambiguous as to whether there is one cue or two. There is also evidence to support the existence of two independent cues. One way to further explore the question is to go beyond low-level estimation tasks and see how the two different types of information affect more complex perceptual processes.

### Frequency of occurrence and duration in music

Musically derived stimuli provide a unique opportunity to study the functional relevance of frequency of occurrence and duration cues in a well-defined context. Frequency of occurrence and durations of individual tones are distributed across the musical surface. These distributions provide information about the probability of tone-events, in that more frequent and longer tones predict tone recurrence. Understanding how these distributions are apprehended, coded and processed can contribute to theoretical debate as to the inter-dependence or separability of the cues.

Three previous and relevant lines of inquiry are that (i) musical compositions in a given style (for present purposes the Western tonal-harmonic system) contain consistent distributions of frequency of occurrence and duration of tones; (ii) these distributions correspond to listeners’ sense of tonality or tonal schema, and; (iii) frequency of occurrence and duration cues may lead to the abstraction of the tonal schema through statistical learning.

#### (i) Distributions within musical compositions

Corpus studies have obtained counts of both frequency of occurrence and duration for each of the chromatic scale tones within the Western tonal-harmonic style. Frequency of occurrence and duration tend to be treated interchangeably in the literature, for, as we shall see below, they provide similar information about tone distributions in a musical idiom. Both counts show a regular pattern of distribution across the scale tones in which some tones are more prominent than others.

Krumhansl [70] reported the frequency of occurrence of pitches from a wide range of Western tonal music, as counted by Knopoff and Hutchinson [71] and Youngblood [72]. These counts correlate highly with the tonal schema of music, or tonality, in the Western harmonic system, as described by music theorists [70, 73–75]. Musicologists posit that music conveys a hierarchic pitch structure such that the most prominent, stable tone within the Western 7-tone diatonic scale is the tonic, the first tone within the scale. At the next level of the hierarchy are the mediant and dominant tones that, along with the tonic, make up the major triad. The rest of the diatonic tones (i.e., the rest of the scale tones) follow at the next level of the hierarchy, finally followed by the nondiatonic tones [76, 77].

#### (ii) Listeners’ sensitivity to tonality in musical contexts

Experimental evidence has demonstrated the psychological reality of musical pitch structure for the Western idiom. Most of the studies supporting this statement have used the probe-tone paradigm developed by Krumhansl and Shepard [78]. In this paradigm listeners are presented with a musical context followed by one of the 12 chromatic scale tones. The listener then rates how well the probe tone fits the context just heard. The musical context is repeated and followed by a different probe tone until ratings for all 12 chromatic tones are obtained. The collection of 12 ratings is termed the probe-tone profile for that context. Across several musical contexts, listeners systematically gave probe tone ratings that are in accord with the hierarchy proposed by music theorists [78–82]. The standardized probe-tone profile, or standardized tonal hierarchy, is the average of probe-tone profiles across many musical contexts that instantiate a sense of Western tonality, across both major and minor keys [82]. The standardized probe-tone profile is considered a measure of the perceived pitch structure and knowledge of the Western tonal-harmonic system (tonal schema—TS) that guides musical perception, recognition and recall.

#### (iii) Listeners’ sensitivity to frequency of occurrence and duration in musical contexts

Listeners’ sensitivity to frequency and duration is thought to promote statistical learning whereby listeners develop an internal model of musical events i.e., a representation of tonal schema, as described in the previous paragraphs [70]. Such a representation appears to be acquired by 5 or 6 years of age, and no formal music training is required [83].

To illustrate the availability of frequency of occurrence and duration as cues to the tonal hierarchy, we have calculated the total duration (D), as well as the frequency of occurrence (F), of tones’ pitches in a representative sample of Western tonal music. Using the Krumhansl-Schmuckler key finding algorithm [70, 84], we have correlated these distributions with the standardized tonal hierarchy (as a measure of tonal schema—TS) based on real listeners’ probe tone ratings for the 12 chromatic tones [70]. It can be seen in Table 1 that frequency of occurrence, total duration, and tonal schema are highly correlated within the Western idiom.

**Table 1:** Correlations between frequency of occurrence (F), total duration (D), and the tonal schema (TS) of the key signature of selected western compositions.

Composer	Piece and date	F–D	F–TS	D–TS
Bach	Invention #3, 1723	.98**	.81**	.91**
Mozart	Sonata #11, 1788	.98**	.83**	.90**
Beethoven	Für Elise, 1808	.98**	.84**	.91**
Schubert	Die Forelle, 1817	.87**	.77**	.84**
Chopin	Nocturne op. 72, 1855	.96**	.69*	.77**
Fauré	Nocturne #4, 1885	.89**	.63*	.79**
Prokofiev	Peter & the Wolf, 1936	.96**	.72**	.66*

Significance (*p < .05, ** p < .01) was calculated for all correlation coefficients based on df = 10. Data compiled by N. A. Smith.

Sensitivity to the surface cues of frequency of occurrence and duration may be responsible for the acquisition of tonal schema through mere exposure to the music. This sensitivity may also predict listeners’ responses to the musical structure of unfamiliar cultures. Western listeners gave the highest probe tone ratings to tones that occurred most often in the unfamiliar musical structures of the Slendro key in the music of Bali [85] and in the music of North India [86]. Lantz, Kim, and Cuddy [87] found that Western participants could differentiate between scale and non-scale tones in traditional Korean music when the former were longer than the latter. Using a modified probe tone paradigm, Raman and Dowling [88] found Western listeners’ probe tone ratings to South Indian music to be influenced by both frequency of occurrence and duration.

Other probe-tone studies have used novel, experimentally constructed, idioms in which the surface cues were not correlated with the tonal idiom [89–91]. For example, Oram and Cuddy [91] directly manipulated the frequency of occurrence of tones within short nontonal sequences. More frequently occurring tones were given higher probe tone ratings than less frequent tones.

Smith and Schmuckler [92] examined duration with short sequences from a 12-tone chromatic distribution. They found that longer tones in sequences received higher probe tone ratings than shorter tones though only when longer durations were applied to the most important tones of a tonal hierarchy. These results suggest that the longer duration only highlights tones to help identify a tonal schema that can then be used to organize the pitch structure of the sequence.

In a second experiment, these authors examined frequency of occurrence. Recognizing that the increasing frequency of occurrence leads to an increase of the total (cumulative) duration, they manipulated frequency of occurrence of tones in two ways. First, they held the duration of every tone constant and allowed total duration to increase as the frequency of occurrence of tones increased. Second, they controlled the total duration of each tone such that the most frequent tones were played for a shorter duration on each occurrence. When the most frequent tones were consistent with the tonal hierarchy, there was an effect of frequency of occurrence but only in the uncontrolled total duration condition. The finding suggested that the effect was due to total duration rather than frequency of occurrence. However, in controlling total duration, the most frequent tones became extremely short. In fact, the authors questioned whether the very shortest of the frequent tones in one condition may have been too short for listeners to even perceive a stable pitch. Regardless whether a stable pitch was perceived, it is likely that such short tones were much less salient than the longer but less frequent tones, perhaps having little effect on the overall perceived pitch structure. Another consideration is whether the frequent tones affected the perceived pitch structure in other ways. In a supplemental analysis, the authors noted that the longer, less frequent tones in the controlled condition implicated two alternative keys. A correlation between the ratings and the standardized tonal hierarchy for each alternative key was not significant. The short, frequent tones appear to have moderated the duration effect that they found in their first experiment as listeners were able to extract the tonal hierarchy from duration.

In conclusion, frequency of occurrence and duration of tones in music are powerful cues that lead to the apprehension of pitch structure. As shown in Table 1, frequency of occurrence and duration correlate highly. Within the musical corpus, there appears to be no route to determining whether the cues reflect a single or dual mechanism. To the best of our knowledge, only Lantz and Cuddy [93] has manipulated both frequency of occurrence and duration within a single sequence to see their effect on perceived pitch structure while avoiding the confound of frequency of occurrence and total duration. The current study is a continuation and expansion on this earlier study.

### Purpose and outline of present research

To this end we employed the probe tone paradigm with an unfamiliar context that contained both frequency of occurrence and duration cues. Both are cues to surface salience. The basic question was whether listeners would respond preferentially to one cue over the other or would treat the cues as a single cue.

Participants rated the fit of probe tones following contexts of randomly ordered tone sequences generated from specific 6-tone tonesets. In each toneset, three tones were biased in their frequency of occurrence. That is, we manipulated their frequency of occurrence to be either the same or greater than the other tones. Likewise, the other three tones were durationally biased. That is, we manipulated their duration to be either the same or greater than the other tones in the toneset. There is a natural confound between frequency of occurrence and total duration. As a tone is heard more frequently, the total duration naturally increases. Therefore, when frequency biased tones occurred more often we ensured that their total duration was the same across all sequence conditions. The six remaining tones from the chromatic scale, designated the non-toneset tones, were not presented in tone sequences but were still used as probe tones.

The sequence conditions were the factorial combination of the durational and frequency manipulations. In Experiments 1 and 2, the three-tone triad biased with duration was perceptually equivalent to the three-tone triad biased with frequency of occurrence (diminished triads and major triads respectively). Thus, we could directly test their salience as frequency of occurrence and duration were directly in opposition to each other within each sequence. In Experiment 3, the cues biased non-equivalent structures within each sequence (a major triad vs. a diminished triad).

Based on the literature described above we expected that non-toneset tones would receive lower ratings than toneset tones. This follows from findings showing that participants are sensitive to both frequency of occurrence and duration cues and both cues may serve to increase salience of information.

Of particular interest however is whether tiered probe-tone ratings, such that tones of one triad would receive higher ratings than the tones of the other triad, would emerge. Such a pattern would indicate that a) frequency of occurrence and duration are likely processed separately, and b) one of the two cues is more salient toward organizing input into higher level perceptual entities. Conversely, if there is no difference in the ratings for the tones of the two triads, it would support the idea that a) they are processed as one cue, and b) thus inseparable when organizing input into higher level perceptual entities.

As described below in the general method, tonesets were chosen to allow analysis of further research questions. In the first experiment, tone sets were constructed to minimize influence of tonality cues. Next, tonesets were constructed to investigate whether participants use tonal schema when tonality cues are available. For the last experiment, tonesets were constructed to investigate whether and how tonality cues may interact with surface regularities, that is directly from the sequences being heard. By quantifying the influence of possible tonal schema, we have an opportunity to illuminate the effect of musical experience on using surface cues to organize input.

## General method of experiments

This study was reviewed and approved by the Queen’s University General Ethics Review Board. Written consent was obtained from each participant prior to each experiment. Three experiments were conducted. Each sought to determine the perceptual salience and separability of frequency of occurrence of tones and duration of tones. They differed only in the degree to which the experimental stimuli, i.e., the melodic sequences, afforded the opportunity to access tonal cues. We first describe the methods and procedures that were common to all experiments, followed by the details and results of the experiments themselves.

### Participants

In each experiment there were 32 participants, 16 musically trained and 16 untrained. Musically trained participants were recruited through posters placed in the Queen’s University School of Music. Each of the trained participants had achieved at least Royal Conservatory of Music (Toronto) grade VIII or equivalent on an instrument or in voice training, with on average 12.5 years of formal music training. Untrained participants were recruited from Psychology undergraduate students. Only a few had taken formal music lessons in the past and none was currently taking lessons or had achieved Royal Conservatory of Music (Toronto) grade V. The age range of the participants was 17 to 45 years (M = 21.7 yrs), with no significant differences between experiments or levels of music training. All participants read a Letter of Information before signing a Consent Form. They were compensated with either a small cash payment or course credit. This procedure was approved by the local research ethics board.

### Sequence conditions

Sequences were created from tonesets of six tones. Three of the tones in each toneset were consistently heard with one of two levels of frequency of occurrence bias (frequency bias or no frequency bias) and were designated the Frequency Triad. The other three tones in each toneset were consistently heard with one of two levels of duration bias (duration bias or no duration bias) and were designated the Duration Triad. Thus, every sequence consisted of a Frequency Triad and a Duration Triad that were directly opposed to each other, competing for perceptual salience.

There were four experimental sequence conditions formed by the crossing of two types of Triad (Frequency vs Duration) and two types of bias (unbiased or biased). For the Frequency Triad, the bias, when applied, involved increasing the frequency of occurrence of each of the three tones by a factor of four. For the Duration Triad, the bias, when applied, involved increasing the duration of each of the three tones by a factor of four.

Sequence conditions were defined by their frequency of occurrence ratio (FOR) and total-duration ratio (TDR). The FOR describes the amount of frequency of occurrence bias given to the Frequency Triad. For example, an FOR of 4:1 (Frequency Triad: Duration Triad) describes a sequence condition in which the Frequency Triad tones occur four times as often as the Duration Triad tones. Similarly, the TDR describes the amount of durational bias given to the Duration Triad. For example, a TDR of 1:4 (Frequency Triad: Duration Triad) describes a sequence condition in which the total duration of Duration Triad tones is four times the total duration of Frequency Triad tones. Table 2 summarizes these sequence conditions along with the frequency of occurrence and duration of the tones of the Frequency Triad and the Duration Triad. In total, there were four sequence conditions.

**Table 2:** Sequence conditions.

		Frequency of occurrence ratio (FOR)	
		1:1 (Frequency Triad: Duration Triad)	4:1 (Frequency Triad: Duration Triad)
Total-duration ratio (TDR)	1:1 (Frequency Triad: Duration Triad)	Condition 1	Condition 2
		*Frequency Triad*: 3 times, 500 ms each, *Duration Triad*: 3 times, 500 ms each	*Frequency Triad*: 12 times, 125 ms each, *Duration Triad*: 3 times, 500 ms each
	1:4 (Frequency Triad: Duration Triad)	Condition 3	Condition 4
		*Frequency Triad*: 3 times, 500 ms each, *Duration Triad*: 3 times, 2000 ms each	*Frequency Triad*: 12 times, 125 ms each, *Duration Triad*: 3 times, 2000 ms each

Sequence conditions described by their Frequency of occurrence ratio (FOR) and Total-duration ratio (TDR) along with frequency of occurrence and duration of the Frequency Triad and the Duration Triad.

Condition 1: an FOR of 1:1 and a TDR of 1:1. Tones of the Frequency Triad and the Duration Triad had the same total duration and occurred equally often;
Condition 2: an FOR of 4:1 and a TDR of 1:1. Tones of the Frequency Triad and the Duration Triad had the same total duration but Frequency Triad tones occurred four times as often as the Duration Triad tones, at a quarter duration of Duration Triad tones;
Condition 3: an FOR of 1:1 and a TDR of 1:4. Tones of the Frequency Triad had the same frequency of occurrence as the tones of the Duration Triad. Tones of the Duration Triad were four times longer in duration than the tones of the Frequency Triad;
Condition 4: an FOR of 4:1 and a TDR of 1:4. Tones of the Frequency Triad occurred four times as often as tones of Duration Triad tones but were much shorter than the tones of the Duration Triad. The duration of the Frequency Triad tones was a sixteenth of that of the of the Duration Triad tones.

**Fig 1:**
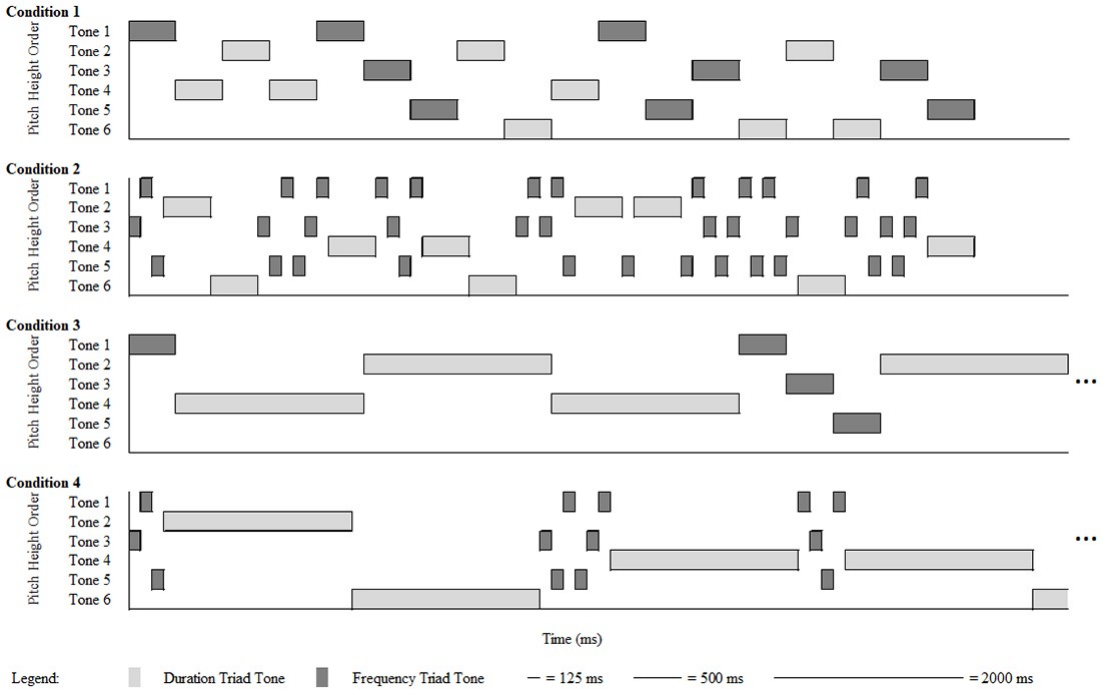
Example sequences. Duration Triad tones are indicated with light grey boxes. Frequency Triad tones are indicated with dark grey boxes. The first 10 seconds are visualized. Continuing sequences are indicated by “…”.

The first 10 seconds of example sequences are visualized in Fig 1 showing the changing frequencies of occurrence and durations for the Frequency Triad and Duration Triad tones across conditions in all experiments. The sequences are hypothetical to represent sequences in all experiments. Although Tones 1, 3, and 5 are biased by frequency of occurrence in these examples, another version of each sequence condition is created in which Tones 1, 3, and 5 are biased by duration as detailed below.

For each experiment, each sequence condition was generated from one of two tonesets, so that the same toneset would not be heard repeatedly. The use of different tonesets ensured that different absolute pitches of tones occurred in different blocks of trials.

Each toneset had two versions. The tones of the Frequency Triad in the first version were the tones of the Duration Triad in the second version, and vice versa. Tonesets and versions were counterbalanced across conditions and within participants.

The tone order for each sequence in all experiments was quasi-random. In order to ensure that the Frequency Triad and the Duration Triad remained perceptually separate, several constraints were placed on the order of tones within a sequence.

1. Adjacent tones in a sequence were always separated in pitch height by at least two semitones to reduce the possibility of assimilation. Bharucha [94–95] reported that a tone might be assimilated into the key (or pitch structure) of the following tone when it is a pitch neighbour, either diatonically or chromatically, of that tone.
2. No tone was allowed to occur in successive serial positions, in order to avoid the possibility that two successive tones would not be considered a single occurrence of twice the given duration rather than two occurrences of the tone.
3. The order of durationally biased tones was irregularly spaced in time to reduce any regular metric structure. A regular metric structure is likely to induce an accent on the durationally-biased tones that may affect their salience [38–39, 96–100]. Although a rhythmic pattern may still occur due to the durational bias, the lack of a regular metric pattern, and the emphasis on pitch in the instructions should reduce the effect of rhythmic accents on the ratings [101].
4. When tonesets contained major triads, sequences did not begin or end with the tonic of either triad. In Western tonal music, the tonic of a piece is often found at the beginning and/or end of the piece and can be a salient cue for participants in perceiving the pitch structure of the piece [70].
5. All tones were in a narrow pitch range C4 (262 Hz) to B4 (494 Hz) to avoid an effect of pitch height on probe tone ratings as much as possible. Thompson and Mor [102] found a pitch height effect in goodness-of-fit ratings across three octaves, with a preference for higher tones.

### Experimental design and procedure

Each condition was presented twice–once with each of the toneset versions–so that there were eight sequence types (4 conditions x 2 versions). The eight sequence types were presented to participants in eight different orders in an 8 x 8 Latin-Square design. With 16 participants in each training group, the Latin Square was completed twice per group. Each of the sequences was presented to participants 14 times in succession. Each presentation was followed by a 1 s pause and then a 1.5 s probe tone. The first two presentations of each sequence type were practice trials with a probe tone randomly chosen from the chromatic set. The 12 experimental trials were each followed by one of the 12 chromatic tones in the range of C4 to B4, the order of which was randomized for each participant. Thus, there were 112 trials in total, with 96 experimental trials.

Musical Instrument Digital Interface (MIDI) files for sequences and probe tones were created with Cakewalk Professional, Version 4.0a [103]. Presentation of stimuli was controlled through the program ’Midiplay’ [104], running on a Zenith Z-200 computer. Sequence tones and probe tones were pure tones generated by a Yamaha TX802 FM tone generator with MIDI input from the Zenith Z-200. The sound was amplified by a Denon PMA-730 amplifier and delivered into a soundproofed booth in which participants heard the sequences through Sennheiser 480 headphones. The participants rated probe tones on a remote Zenith computer terminal within the booth. Sound pressure level was set to a comfortable level by each participant, approximately 55 to 70 dB SPL.

Participants were tested individually. They were told they would hear a short sequence of tones, followed by a pause and then a final tone. They were asked to rate the final probe tone as to how well the tone seemed to ’fit’ within the sequence they had just heard on a scale from ’1—fits very poorly’ to ’7—fits very well’. They were asked to try to use the entire scale as they rated the probe tones. The rating scale was displayed on the remote terminal whenever the participant was to make a rating. All responses for the trials were typed into the remote terminal and were recorded directly into a data file on the Zenith Z-200. Midiplay ignored responses not in the range of one to seven and the participants were prompted to re-enter the rating from the correct range. Afterwards, participants completed a questionnaire detailing their musical background and demographic information, were debriefed, and payment, if any, was made. The entire procedure was completed in approximately 90 minutes.

### Statistical analysis

Probe tone ratings were averaged for each category of tone, the Duration Triad tones, the Frequency Triad tones, and non-toneset tones, yielding three averaged ratings per participant for each of the sequence types. These averaged ratings were submitted to a five-factor mixed ANOVA followed by contrasts on the experimental effects of interest. The between-subjects factor was training (2 levels). The four within-subjects factors were Tone Category (3 levels), TDR (2 levels), FOR (2 levels), and Version (2 levels).

All three experiments report analyses of unstandardized data. Similar analyses were conducted after the data had been standardized for each participant with respect to their mean rating. In all three experiments, results from standardized data showed the same pattern of results as the unstandardized data.

In order to estimate the replicability of our results, we further calculated Bayes factors using the Bayesian statistics package in JASP [105] as done in recent research in our area [106]. Bayes factors, *BF*_01_, indicate how much more likely the results are given the null hypothesis compared to the alternative hypothesis. The inverse of Bayes factors, *BF*_10_, indicate how much more likely the results are given the alternative hypothesis compared to the null hypothesis. We feel the latter is a more intuitive way of showing the magnitude of the effect since larger *BF*_10_ indicate stronger support for the alternative hypothesis. As a rule of thumb, *BF*_10_ larger than *BF*_10_ = 3 indicate positive or substantial support for the alternative hypothesis, and *BF*_10_ larger than *BF*_10_ = 150 indicate very strong or decisive support for the alternative hypothesis. By contrast, *BF*_10_ lower than *BF*_10_ = 3 indicate weak or anecdotal support for the alternative hypothesis [107].

### Individual experiments

Across three experiments, the availability of cues to tonality was varied, both in the construction of the triadic patterns and in the toneset formed by the interleaving of the triad tones. The three tones of the triads formed either a tonally strong pattern, meaning that the triad reliably instantiated the tonal schema of a major key, or a tonally weak pattern, meaning that the triad did not instantiate the tonal schema of any major or minor key. The strong pattern was the major triad (e.g., C-E-G) and the weak pattern was the diminished triad (e.g., B-D-F).

The major triad, the first, third, and fifth steps of the major scale, contains theoretically simple harmonic ratios among the tones, and is the basis of Western harmonic music theory [108, 109]. Psychologically, it yields an unambiguous sense of major key [70, 79]. The diminished triad, formed by the seventh, second, and fourth steps of the major scale, contains theoretically complex ratios among the tones. It is perceptually unstable with respect to a sense of key [79, 80].

Comparison of the results across the three experiments allowed assessment of the influence of tonal cues on the pick-up of surface cues of duration and frequency of occurrence.

## Experiment 1

Triads and tonesets of Experiment 1 were constructed to minimize the influence of tonality cues. The tones used for the Duration Triad and the Frequency Triad both formed diminished triads from unrelated keys. Neither triad individually provided tonal information nor did the toneset formed by combining the two triads. To assess numerically the possibility that the toneset could provide tonal information, correlations were conducted between a vector representing toneset tones (coded ‘1’) and non-toneset tones (coded ‘0’) and the standardized probe tone profile for all major and minor keys [82]. None of the correlations was significant, *p*s > .100.

### Stimulus contexts

The diminished triads for toneset I were “C-E♭-G♭” and “D-F-A♭”. The diminished triads for toneset II were “C♯-E-G” and “D♯-F♯-A”. Thus, the tonesets for experiment 1 were: I. “C-D-E♭-F-G♭-A♭” (conditions 1 and 3) and II. ‘‘C♯-D♯-E-F♯-G-A” (conditions 2 and 4).

### Results and discussion

**Fig 2:**
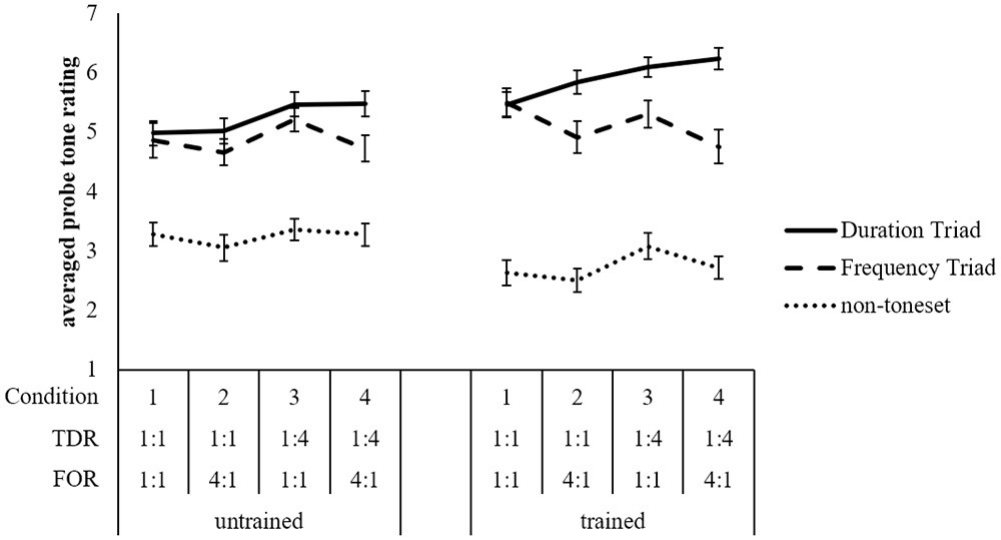
Probe tone ratings in Experiment 1. Averaged probe tone ratings in Experiment 1 for each tone category collapsed across version for both levels of training. Error bars indicate standard error of the mean.

Fig 2 shows the averaged probe-tone ratings for each tone category (y-axis) across each stimulus condition, with untrained and trained groups shown separately. Data are collapsed across Version as there were no significant effects involving this factor. The averaged probe tone ratings for the Duration Triad are shown laying on a solid line; for the Frequency Triad laying on a dashed line; and for non-toneset tones on a dotted line. The data are shown separately for trained participants and untrained participants.

For both levels of training, a three-tiered pattern emerges in Fig 2. Toneset tones were rated higher than non-toneset tones, and Duration Triad tones were rated higher than Frequency Triad tones, except in the Condition 1 in which all tones were isochronous. The main effect of tone category was significant, *F*(2, 60) = 231.36, *p* < .001, *BF*_10_ > 150. Post hoc tests using Bonferroni correction for multiple comparisons between tone categories revealed significant differences between all tone categories. Specifically, Duration Triad tones were rated higher than Frequency Triad tones, and Frequency Triad tones were rated higher than non-toneset tones, *p*s < .001. The distinction between categories was greater for trained participants than untrained participants, reflected by a significant interaction between tone category and training, *F*(2, 60) = 13.30, *p* < .001, *BF*_10_ > 150.

Further inspection of Fig 2 reveals that the tiered pattern becomes more evident as FOR and TDR are increased, yielding significant interactions between tone category and FOR, *F*(2, 60) = 9.79, *p* < .001, *BF*_10_ = 24.70, and tone category and TDR, *F*(2, 60) = 5.91, *p* < .001, *BF*_10_ = 3.88. No other interactions involving tone category were significant, *p*s > .05.

We followed up the significant interaction between tone category and FOR by examining the presence of this effect for the Duration Triad and the Frequency Triad, and Frequency Triad and non-toneset tones. For the Duration Triad and the Frequency Triad, the interaction between tone category and FOR was significant, *F*(1,30) = 15.05, *p* = .001, *BF*_10_ > 150, with the Duration Triad receiving higher ratings at both levels of FOR, though more so when FOR = 4:1, *F*_FOR 1:1_(1,30) = 10.85, *p* = .003, and *F*_FOR 4:1_(1,30) = 59.11, *p* < .001. To be clear, the preference for the Duration Triad is greatest when the Frequency Triad tones are most frequent, although played for their shortest duration. For the Frequency Triad and non-toneset tones, the interaction between tone category and FOR was not significant, *F*(1,30) = 1.83, *p* = .186, *BF*_10_ = 0.61.

We followed up the significant interaction between tone category and TDR in the same manner. For the Duration Triad and the Frequency Triad, the interaction between tone category and TDR was significant, *F*(1,30) = 12.36, *p* = .001, *BF*_10_ = 44.99, with the Duration Triad receiving higher ratings at both levels of TDR, though more so when TDR = 1:4, *F*_TDR 1:1_(1,30) = 12.46, *p* = .001, and *F*_TDR 1:4_(1,30) = 63.82, *p* < .001. For the Frequency Triad and non-toneset tones, the interaction between tone category and TDR was significant but the corresponding Bayes factor did not warrant follow-up, *F*(1,30) = 4.24, *p* = .048, *BF*_10_ = .381.

The results of our repeated ANOVAs were supported by Bayesian statistics. *BF*_10_ indicated positive or substantial to very strong or decisive support for the alternative hypotheses. These results suggest that in the absence of cues from tonal schema, listeners systematically created a 3-tiered hierarchy of pitch structure with Duration Triad tones at the top of the hierarchy, Frequency Triad tones next in the hierarchy and non-toneset tones at the bottom of the hierarchy. A clear difference exists between ratings for toneset tones and non-toneset tones, indicating that the longer tones and the more frequent tones both affected the perception of pitch structure. Whenever the duration bias was applied, Duration Triad tones were rated higher than Frequency Triad tones. More surprisingly, Duration Triad tones also were rated higher when the frequency of occurrence bias was applied to Frequency Triad tones. This indicates that participants primarily relied on duration cues to pitch structure, and that duration cues were stronger than frequency of occurrence cues. One possibility is that the higher relative duration of Duration Triad tones disadvantaged Frequency Triad tones. That is, listeners relied on longer tones to organize the pitch structure. Recall that in order to control for the total duration with which each Frequency Triad tone was sounded, the quadruple increase in frequency of occurrence was accompanied by a corresponding reduction in the duration of each Frequency Triad tone. The 2000 ms Duration Triad tones overwhelmed the 125 ms or 500 ms Frequency Triad tones. However, it was not the case that the shortest tones were perceptually ignored, as they still retained a preference over non-toneset tones. It is possible that Frequency Triad tones would have received even lower ratings had they occurred less often than they did in the sequences. In that sense, both duration and frequency of occurrence affected the perceived pitch structure of the sequences.

Of note is the clearer pattern given by trained participants. This could be due to several reasons. First, it is possible that trained participants are simply more attentive. It is also possible that trained participants have acquired a strategy to focus on surface cues when unable to access tonal schema [91].

## Experiment 2

Triads of Experiment 2 were constructed to provide tonality cues; thus, results from Experiment 2 should reveal whether participants use duration and frequency of occurrence cues to form a pitch structure when they have access to tonal schema. The Duration Triad and the Frequency Triad were both major triads. The tonesets, i.e., tones of both triads together, did not correlate significantly with the standard probe tone profiles of any key, making it unlikely that the stimulus sequence on the whole implied any key, *p*s > .300.

### Stimulus contexts

The major triads for toneset I were “C-E-G” and “F♯-A♯-C♯”. The major triads for toneset II were “C♯-E♯-G♯” and “G-B-D”. Thus, the tonesets for experiment 2 were: I. “C-C♯-E-F♯-G-A♯” (conditions 1 and 4) and II. “C♯-D-E♯-G-G♯-B” (conditions 2 and 3).

### Results and discussion

**Fig 3:**
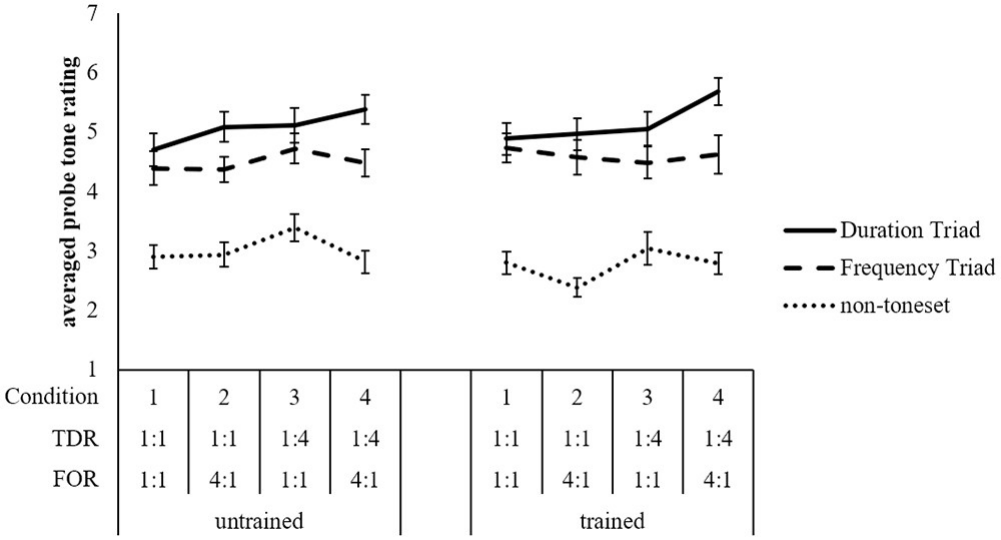
Probe tone ratings in Experiment 2. Averaged probe tone ratings in Experiment 2 for each tone category collapsed across version for both levels of training. Error bars indicate standard error of the mean.

Results are shown in Fig 3, with the same format as Fig 2. Once again for both levels of training, a three-tiered pattern emerges. Toneset tones were rated higher than non-toneset tones, and the Duration Triad tones were rated higher than the Frequency Triad tones, except in Condition 1 in which all tones were isochronous.

The main effect of tone category was significant, *F*(2, 60) = 146.48, *p* < .001, *BF*_10_ > 150. Post hoc tests using Bonferroni correction for multiple comparisons between tone categories revealed significant differences between all tone categories. Specifically, Duration Triad tones were rated higher than Frequency Triad tones, and Frequency Triad tones were rated higher than non-toneset tones, *p*s < .001. There was no significant interaction between tone category and training, *F*(2, 60) = 1.17, *p* > .05, *BF*_10_ = 1.22.

Additionally there was a significant interaction between tone category and FOR, *F*(2, 60) = 8.37, *p* < .001, *BF*_10_ = 70.87. No other interactions involving tone category were significant, *p*s > .05. We followed up the significant interaction between tone category and FOR by examining the presence of this effect for the Duration Triad and the Frequency Triad, and the Frequency Triad and non-toneset tones. For the Duration Triad and the Frequency Triad, the interaction between tone category and FOR was significant, *F*(1,30) = 6.20, *p* = .019, *BF*_10_ = 10.31, with the Duration Triad receiving higher ratings at both levels of FOR, though slightly more so when FOR = 4:1, *F*_FOR 1:1_(1,30) = 22.09, *p* < .001, and *F*_FOR 4:1_(1,30) = 26.29, *p* < .001. For the Frequency Triad and non-toneset tones, the interaction between tone category and FOR was not significant, *F*(1,30) = 1.52, *p* = .228, *BF*_10_ = 0.33.

The results of our repeated ANOVAs were supported by Bayesian statistics. *BF*_10_ indicated positive or substantial to very strong or decisive support for the alternative hypotheses. These results suggest that in the presence of tonal cues, participants still used both duration and frequency of occurrence cues to form a pitch hierarchy. A clear difference is shown between toneset tones and non-toneset tones, indicating that participants did indeed abstract frequency of occurrence cues. That Duration Triad tones were rated higher than Frequency Triad tones once again indicates duration cues were more salient than frequency of occurrence cues. Duration Triad tones were rated higher even when the frequency of occurrence bias was applied to the Frequency Triad. One possibility here is that the greater relative duration of Duration Triad tones may have disadvantaged frequency cues.

In contrast to Experiment 1, there was no effect of training. This is an argument against the idea that trained participants are simply more attentive participants. Instead, it seems that the absence of tonal cues in Experiment 1 may have encouraged trained participants to focus on surface cues.

## Experiment 3

The results from Experiments 1 and 2 revealed a stronger influence of duration over frequency of occurrence cues when the triads were of equal tonal strength. In Experiment 3, only one of the triads provided tonal cues; either the Duration Triad was a major triad and the Frequency Triad was a diminished triad or vice versa. Thus, we assigned tonal strength either in correspondence with or pitted against the perceptual cues (Duration Triad = major triad or Frequency Triad = major triad respectively). The toneset comprising both triads correlated significantly with the standardized probe tone profile of the major key associated with the major triad, *r*(10) = .66, *p* < .05.

### Stimulus contexts

The major triad for toneset I was “C-E-G” and the diminished triad for toneset I was “D-F-A♭”. The major triad for toneset II was “E♭-G-B♭” and the diminished triad for toneset II was “F-G♯-B”. Thus, the tonesets for experiment 3 were: I. “C-D-E-F-G-A♭” (conditions 1 and 4) and II. “E♭-F-G-G♯-B♭-B” (conditions 2 and 3).

### Results and discussion

**Fig 4:**
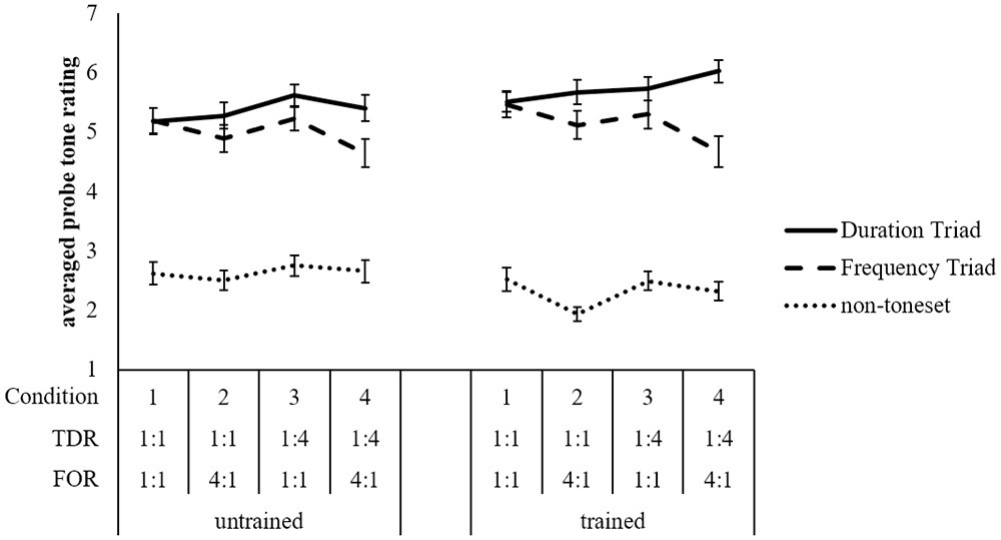
Probe tone ratings in Experiment 3. Averaged probe tone ratings in Experiment 3 for each tone category collapsed across version for both levels of training. Error bars indicate standard error of the mean.

**Fig 5:**
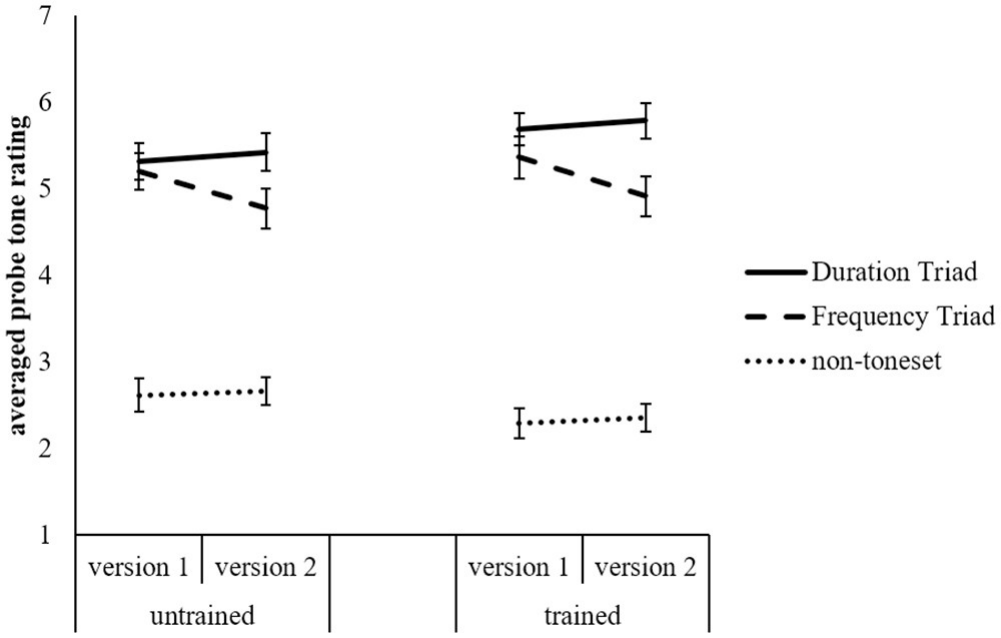
Probe tone ratings in Experiment 3 across conditions. Averaged probe tone ratings in Experiment 3 for each tone category collapsed across conditions for both levels of training. Error bars indicate standard error of the mean.

Results are shown in Fig 4 in the same format as in Figs 2 and 3. An additional figure, Fig 5, shows averaged probe tone ratings for both toneset versions (Duration Triad = diminished triad, vs Duration Triad = major triad) and for trained and untrained participants collapsed across conditions. In Fig 4, it can be seen that once again for both levels of training, a three-tiered pattern emerged. Toneset tones were rated higher than non-toneset tones, and Duration Triad tones were rated higher than Frequency Triad tones, except in Condition 1 in which all tones were isochronous.

The main effect of tone category was significant, *F*(2, 60) = 354.34, *p* < .001, *BF*_10_ > 150. Post hoc tests using Bonferroni correction for multiple comparisons between tone categories revealed significant differences between all tone categories. Again, Duration Triad tones were rated higher than Frequency Triad tones, and Frequency Triad tones were rated higher than non-toneset tones, *p*s < .001. The distinction between categories was greater for trained participants than untrained participants, reflected by a significant interaction between tone category and training, *F*(2, 60) = 4.51, *p* = .015, *BF*_10_ > 150.

Additionally there was a significant interaction between tone category and FOR, *F*(2, 60) = 12.80, *p* < .001, *BF*_10_ = 46.76, that was further qualified by a significant interaction between tone category, FOR, and training, *F*(2, 60) = 7.76, *p* < .001, *BF*_10_ = 7.96. The separation between the Duration Triad and the Frequency Triad was clearer at an FOR of 4:1 compared to an FOR of 1:1, *F*_FOR 1:1_(1,30) = 7.06, *p* = .013, and *F*_FOR 4:1_(1,30) = 35.18, *p* < .001, especially for trained participants. There was also a significant interaction between tone category and TDR, *F*(2, 60) = 7.24, *p* = .002, *BF*_10_ = 3.95. The separation between the Duration Triad and the Frequency Triad was clearer at a TDR of 1:4 compared to a TDR of 1:1, *F*_TDR 1:1_(1,30) = 6.94, *p* = .013, and *F*_TDR 1:4_(1,30) = 38.02, *p* < .001, with the opposite pattern for the separation between the Frequency Triad and non-toneset tones, *F*_TDR 1:1_(1,30) = 388.73, *p* < .001, and *F*_TDR 1:4_(1,30) = 228.04, *p* < .001. Furthermore, there was a significant interaction between tone category and version, *F*(2, 60) = 11.35, *p* < .001, *BF*_10_ > 150, such that the tiered pattern was clearer when the Duration Triad was the major triad and the Frequency Triad was the diminished triad compared to when the Duration Triad was the diminished triad and the Frequency Triad was the major triad (Fig 5).

The results of our repeated ANOVAs were supported by Bayesian statistics. *BF*_10_ indicated positive or substantial to very strong or decisive support for the alternative hypotheses. Thus, the main finding of a three-tiered pattern in Experiments 1 and 2 was once again replicated. Although participants were responsive to both surface cues, the duration cue was more salient. Of particular interest from Experiment 3 is the finding that the three-tiered pattern was clearer when the Duration Triad was a major triad—that is, when tonality and surface cues worked together, the tiered pattern was stronger. In contrast, when tonality and surface cues were pitted against each other, the Duration Triad and the Frequency Triad were scarcely differentiated by their probe tone ratings. Toneset tones, however, still received higher probe- tone ratings than non-toneset tones.

## Comparison of experiments

**Fig 6:**
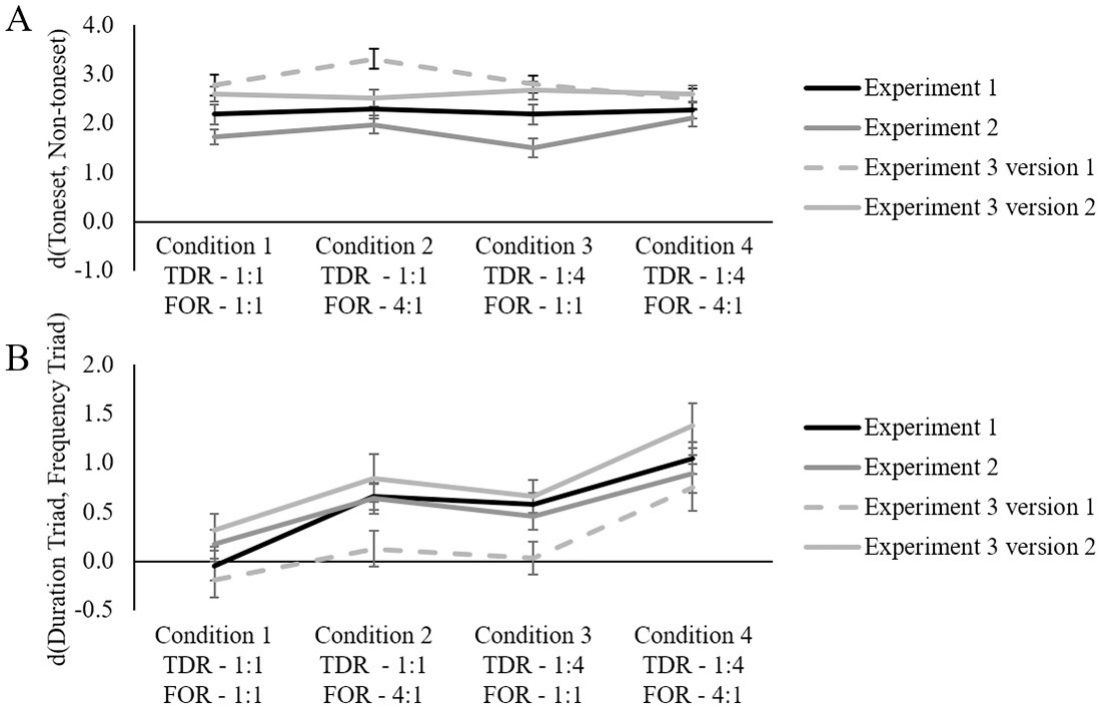
Comparison of Experiments. A: averaged difference between ratings for toneset and non-toneset tones for each condition (Toneset minus Non-toneset), B: averaged difference between ratings for Duration Triad and Frequency Triad tones for each condition (Duration Triad minus Frequency Triad). Error bars indicate standard error of the mean.

Fig 6A and 6B show the difference between averaged ratings for toneset and non-toneset tones, and the difference between averaged ratings for Duration Triad and Frequency Triad tones for each condition in each experiment collapsed across trained and untrained participants (for a summary of the sequence conditions see Table 2). Given the interaction of version and tone category in Experiment 3, the ratings are shown separately for the two versions.

The four panels of Fig 6A show that the averaged difference in ratings between toneset and non-toneset tones (Toneset minus Non-toneset) did not vary systematically across conditions within each experiment. The four panels of Fig 6B, however, do show a systematic difference in ratings across conditions between Duration Triad and Frequency Triad tones (Duration Triad minus Frequency Triad). This systematic difference is apparent for Experiments 1 and 2, and also for the version of Experiment 3 in which the Duration Triad was the major triad. Fig 6B shows that, for these experimental conditions, the difference between ratings for Duration Triad and Frequency Triad tones was greatest in Condition 4, followed in order by Condition 2, Condition 3, and Condition 1. For the version of Experiment 3 in which the Duration Triad was the tonally weak diminished triad (extreme right hand panel), participants did not differentiate between Duration Triad and Frequency Triad tones in Conditions 2 and 3. For this version, only in Condition 4 was the durational advantage of the Duration Triad sufficient to facilitate a preference for the Duration Triad over Frequency Triad. It would appear that durationally biasing the unstable diminished triad led to a reduction in the ability of listeners to abstract the durational information.

Results in Fig 6B are supported by a significant interaction of Version, Tone Category and Experiment, *F*(4, 180) = 4.04, *p* < .001, *BF*_10_ > 150, in which ratings from all three experiments were analyzed in a six-factor mixed ANOVA with between-subjects factors Training (2 levels) and Experiment (3 levels), and within-subjects factors Tone Category (3 levels), TDR (2 levels), FOR (2 levels), and Version (2 levels).

Follow-up tests show that the separation between Duration Triad and Frequency Triad was significant in each experiment and for each version, *F*_Exp 1 V 1_(1, 90) = 17.44, *p* < .001, *BF*_10_ > 150, and *F*_Exp 1 V 2_(1, 90) = 31.90, *p* < .001, *BF*_10_ > 150, *F*_Exp 2 V 1_(1, 90) = 34.63, *p* < .001, *BF*_10_ > 150, and *F*_Exp 2 V 2_(1, 90) = 10.90, *p* < .001, *BF*_10_ = 17.10, except for the version of Experiment 3, in which the Duration Triad was the diminished triad, *F*_Exp 3 V 1_(1, 90) = 2.12, *p* = .149, *BF*_10_ = 0.62, and *F*_Exp 3 V 2_(1, 90) = 55.98, *p* < .001, *BF*_10_ > 150.

Further follow-up tests examining the separation of Frequency Triad and non-toneset tones show that it was significant for all experiments and all versions, *F*_Exp 1 V 1_(1, 90) = 132.41, *p* < .001, *BF*_10_ > 150, and *F*_Exp 1 V 2_(1, 90) = 175.02, *p* < .001, *BF*_10_ > 150, *F*_Exp 2 V 1_(1, 90) = 73.11, *p* < .001, *BF*_10_ > 150, and *F*_Exp 2 V 2_(1, 90) = 128.94, *p* < .001, *BF*_10_ = 17.10, *F*_Exp 3 V 1_(1, 90) = 257.76, *p* < .001, *BF*_10_ > 150, and *F*_Exp 3 V 2_(1, 90) = 226.52, *p* < .001, *BF*_10_ > 150.

The influence of the stability of the major triad can be examined in two ways. First, through comparing data from Experiment 1 to data from Experiment 2 we can examine what happens when the triads are either stable or unstable perceptual elements. As shown in Fig 6A and 6B, such a comparison reveals slightly smaller overall differences for Experiment 2 compared to Experiment 1, supported by a significant interaction between Experiment and Tone Category, *F*(2,120) = 3.42, *p* = .036, *BF*_10_ > 150 (see results sections for each experiment regarding experiment specific test-statistics). This finding again suggests an influence of the tonal strength of the major triad. Perhaps the presence of two strongly but unrelated tonal triads (known as bitonality [84]) distracts from attention to the surface cues. In other words, perhaps attention to surface cues is facilitated when the context is nontonal, and this facilitation is more effective with music training.

Second, through analyzing the effect of version in Experiment 3 we can examine what happens when tonal strength is either in correspondence with or pitted against the salience of perceptual cues. As we have shown above in the report of Experiment 3, the difference in tonal strength between the major and diminished triad is such that, if the durational cue is paired with weak tonal strength, the durational advantage disappears.

Another possible influence of major triads is assimilation of probe tone ratings to a tonal hierarchy. To examine this possibility for each experiment and each condition, we regressed the full set of 12 probe tone ratings, averaged across participants for each chromatic tone, on two types of predictors: One type corresponded to the cue-based hierarchies, or perceptual-cue hierarchies, shown across the three Experiments. The perceptual cue predictor contained ordinal values where Duration Triad tones were coded “2”, Frequency Triad tones were coded “1’ and non-toneset tones were coded “0”. The other type of predictor corresponded to the key profiles implied by the triads or by the entire toneset (tonal hierarchies with values as quantified by Krumhansl [70]). In all regression analyses the perceptual-cue hierarchy predictor explained such large amounts of the variance (ranging from 84.8% to 96.7%) that it is not surprising that we did not find meaningful contributions of tonal hierarchy predictors.

Thus, across three experiments the role of the major triad has not been to engage implied tonality across all probe tones. Rather, the effect is through its inherent tonal strength.

## General discussion

The purpose of the three experiments was to investigate the role of frequency of occurrence and duration in the organization of auditory input into perceptual structures. We did so through the lens of music perception by examining how participants respond to these surface cues in tone sequences, artificially generated to accommodate certain musical principles. Our results strongly suggest that participants were able to derive pitch structures directly from the surface–that is, from the distributional properties of tones in a sequence without reference to cognitive (tonal) schema.

In all three of our experiments, varying in the degree to which the experimental stimuli afforded the opportunity to access the musical cue of tonality, probe-tone ratings from both trained and untrained participants revealed a three-tiered hierarchy in perceived pitch structure. Longer tones formed the top tier of the hierarchy, more frequent tones formed the second tier, and tones that did not occur in the sequence formed the lowest tier of the hierarchy. The distinction between these levels was influenced by frequency bias and duration bias manipulations. Although duration dominated frequency of occurrence, especially when long tones were heard against the shortest most frequent tones, listeners were sensitive to both duration and frequency of occurrence information in each sequence.

### Two regularities to which we are sensitive—Frequency of occurrence and duration of events

We found that tones that were relatively long in duration perceptually dominated relatively shorter, but more frequent, tones. The duration effect was consistent in every condition in which tones were durationally biased, for both untrained and trained listeners. The findings support a similar finding in Smith and Schmuckler [92] who attributed their duration effect to total (cumulative) duration. However, our results show that there is more than just a total-duration effect.

In Conditions 3 and 4 in each Experiment, the Duration Triad tones were all the same absolute duration and therefore had the same total duration (TDR = 1:4 in both conditions). Frequency Triad tones, though, increased in frequency of occurrence from Condition 3 (FOR = 1:1) to Condition 4 (FOR = 4:1). The total duration for the Frequency Triad was held constant in the two Conditions and yet there was a larger differential effect in ratings in favor of the Duration Triad in Condition 4 in which Frequency Triad tones increased in frequency. This is also seen for the similar comparison of Conditions 1 and 2. As shown in Fig 6B, as the frequency of occurrence increased for the Frequency Triad, there was a larger preference in the ratings for the Duration Triad over the Frequency Triad.

The role of frequency of occurrence in the perception of pitch structure appears to be a complex one. The Frequency Triad received significantly higher ratings than non-toneset tones in every condition across the three experiments. Simply occurring in the sequence affected the ratings. However, increasing the frequency of occurrence did not lead to an increase in ratings for the Frequency Triad, but increased the preference for the Duration Triad over the Frequency Triad. The effect can be explained in that holding the total duration constant when increasing the FOR leads to a natural confound with relative duration—as FOR increased, the durations of Frequency Triad tones decreased relative to Duration Triad tones. There may still have been some positive effect of frequency of occurrence. In the cases with the Frequency Triad tones at their shortest, we suspect that ratings would have been much lower for Frequency Triad tones had they only occurred two or three times rather than 12 times in the sequence. However, that is an empirical question that we cannot answer with current data. Caution should be taken in interpreting the results of frequency-of-occurrence experiments in which neither total duration nor relative duration have been controlled or examined.

The sensitivity to both frequency of occurrence and duration information shown here is in line with previous research showing that people are adept at estimating the frequency of occurrence and duration of events [9–16], and that people use both in organizing perceptual input [24–26, 35–37]. The effect of duration could have been moderated by accents in a rhythmic pattern created by the long durations. Researchers [100, 101] have found that strong beats in a regular metric pattern could influence probe-tone ratings. However, instructions that stressed pitch in Prince et al. [101] greatly reduced the effect of metric position on the ratings. An overwhelming proportion of the variance in ratings was accounted for by pitch information compared to metric position. Our sequences were created such that long tones did not stress a regular metric pattern. Given that our instructions focused on pitch, we are confident that our patterns of ratings reflect listeners’ perceived pitch structures without undue influence of a metric accent. Nonetheless, long tones can create an accented, non-metric rhythm [110] that may have affected probe-tone ratings. We suggest that duration increases the salience of longer tones and that it is the increased salience that creates the accented rhythmic patterns. Perhaps future research could try to tease apart duration and accents created by long durations.

### One or two cues?

Our results imply that frequency of occurrence and duration are two separate cues rather than one. A single magnitude accumulator mechanism, such as that proposed by some researchers [41–44] cannot account for the differential ratings for the Duration and Frequency Triads. Our sequences were created with a duration bias on some tones interspersed with a frequency of occurrence bias on different tones within the same sequence. A single magnitude accumulator would not be able to track durationally-biased tones at the same time as it tracks the frequency-biased tones. A single magnitude accumulator could have tracked the accumulated duration of all six tones in the toneset, yet if that had happened, all six tones in the toneset should have received approximately equal ratings. The Duration Triad and the Frequency Triad have the same total duration in Conditions 1 and 2 so if there is a single-mechanism magnitude accumulator, there should be no difference in ratings for these triads. Fig 6B clearly shows the significant advantage of the Duration Triad in Condition 2. Further, the differences in ratings between the Duration Triad and the Frequency Triad should be the same in Conditions 3 and 4 given a single-mechanism magnitude accumulator, yet they clearly are not, with the Duration Triad having a much larger advantage in Condition 4.

The notion of two separable auditory cues is convergent with conclusions from many non-auditory studies cited in the introduction to this paper, studies ranging from animal discrimination tasks to human magnitude estimation and human interference paradigms. Our finding that duration cues are perceptually more salient than frequency of occurrence cues has also been reported elsewhere [69, 92] but sometimes the reverse has been found with frequency of occurrence dominating duration [65, 66]. Future research should direct efforts to determining the circumstances in which one cue dominates the other, whether it is the modality under study (auditory or visual), the nature of the experimental task (abstraction of cues or direct magnitude estimation), or the participant sample. An examination on the effects of total and relative duration with respect to frequency of occurrence is also warranted. Moreover, to explore the two-cue interpretation further, studies should be directed toward establishing in which ways the neural overlap seen in previous research can support these distinct mechanisms [49–57].

### Frequency of occurrence and duration in music

As outlined in the introduction, music provides an excellent platform from which to study perceptual sensitivity and attention to frequency of occurrence and duration of tones. Musical compositions contain consistent distributions of the two cues in a manner dictated by culture and by style. These cues are available at the musical surface and, according to music theorists, signal the pitch and rhythmic structure of the composition. With respect to pitch organization, a first question is whether music experience and/or training are necessary prerequisites for cue abstraction. A second is whether such abstraction is restricted to the musical distributions with which listeners are familiar. Our results strongly suggest that the answer to both questions seems to be ‘no’.

Our findings are in line with a growing body of research showing that participants in general can derive pitch structure directly from distributional regularities at the musical surface, whether those are unfamiliar nontonal distributions [89–91] or statistical distributions at the surface of non-Western music even while being unable to extract the underlying tonal structures from the unfamiliar music [85–87, 111, 112].

The role of music exposure can be pursued further. With exposure to music, listeners are thought to internalize the regularities contained in the distribution to form mental schema [70, 83]. That is to say, the close relation of distributional properties of Western-harmonic music to its tonal schema (see Table 1) suggests that people may gain the tonal schema through repeated exposure and statistical learning.

Tone sequences generated from musical constraints may therefore be constructed to be convergent or divergent with a given schema. The use of these musically derived tone sequences allows us to ask questions about the possible role of schema in the perceptual ability to abstract cues from the musical surface.

Under present conditions, in which the surface distribution was uncorrelated with tonal schema, duration cues were more salient than frequency of occurrence cues, confirming findings by Smith and Schmuckler [92]. In both sets of studies, the availability of tonal structure influenced participants, but in different ways, as will be described further.

Using the probe-tone technique, Smith and Schmuckler [92] also examined sensitivity to duration and frequency of occurrence in algorithmically composed pitch sequences. Similar to present findings, they found that participants were able to derive a sense of pitch structure based on the duration of the tones in the sequences (Experiment 1 in [92]). They were also able to derive a sense of pitch structure based on frequency of occurrence, but only when frequency varied in accordance with tone duration, i.e., without controlling for the total duration of each pitch (Experiment 2 in [92]). In contrast to present findings, they found that durational cues were effective only if they were distributed across the context in a manner consistent with musical schema, with values corresponding to the tonal hierarchy (i.e., longest duration for a tonic, next longest duration for the members of the tonic triad, next longest duration for other scale tones, shortest duration for nonscale tones).

The focus of Smith and Schmuckler’s work, however, was to examine duration and frequency of occurrence separately and individually. Only in Exp. 2, in the controlled total duration condition did they inadvertently put the two cues in opposition to each other. Our full intention was to see the effect of the two cues together in a factorial manner. Their contexts were random orders of the 12-tone chromatic scale; the cues were applied to tones within the distribution either in accordance with the Western tonal hierarchy (hierarchical condition) or applied at random (nonhierarchical condition). Smith and Schmuckler concluded that only for the hierarchical condition did listeners’ ratings show an effect of duration.

In the present study, listeners had to differentiate six tones with only two different durations, the duration assigned to the tones of the Duration Triad and the duration assigned to the tones of the Frequency Triad. In the Smith and Schmuckler study, listeners had to differentiate 12 different durations that were not multiples of each other, and organize them into a pitch hierarchy. Their task was therefore more difficult and complex, requiring more information to be processed than the present study. To deal with such circumstances, the authors suggest that the pattern of durations “acted as a cue to allow listeners to recognize a tonal hierarchy and thus, to apprehend the musically important relations inherent in the structure of this hierarchy […] the pattern induced a particular analytical set on the perception of the individual tones” (p. 275).

The role of tonal schema in the present experiments was present but quite subtle. Note that the Duration Triad and the Frequency Triad were never supported in the 6-tone context by the tonal hierarchy to which the triad belonged. The triads were selected, however, to be tonally weak or tonally strong—that is, they differed in their strength of activation of a tonal centre and a tonal hierarchy. When comparing results from Experiment 1 and Experiment 2, we suggested that the availability of tonal schema may have distracted musically trained participants from the surface cues. Musically trained participants in our experiments showed in general greater differentiation of tone categories except in Experiment 2, in which both the Duration Triad and the Frequency Triad were major triads, i.e., tonally strong elements. Thus, the activation of tonal schema by both triads may have encouraged participants to place less importance on surface cues. In the absence of tonal cues musically trained participants may be primed to pay particular attention to surface cues, explaining their added differentiation seen in Experiments 1 and 3. This possibility could be investigated in greater detail in the future.

In Experiment 3, we manipulated tonal strength by placing it in correspondence with or pitting it against the perceptual cues. When the Duration Triad was the tonally weaker triad participants were unable to distinguish between Duration Triad and Frequency Triad tones. Only in Condition 4 was the durational advantage of the Duration Triad sufficient to overcome the tonally weaker makeup of the triad. Our conclusions, therefore are the same conclusions as Smith and Schmuckler: when duration and tonal schema work in concert we get stronger differentiation and organization. For Smith and Schmuckler, that occurred when duration was varied according to the tonal hierarchy. For the present study, it occurred when duration bias was applied to the tonally strong element in Experiment 3.

Using musical stimuli also allows us to investigate the influence of available perceptual entities on how frequency of occurrence and duration cues influence the formation of a new perceptual entity. As laid out in our comparison of results of all three experiments, the major triad as a tonally strong element did not engage implied tonality across all tones. Its inherent strength when assigned to the Frequency Triad and pitted against the weaker strength of the Duration Triad in one version of Experiment 3 seemingly prevented participants from distinguishing between Duration Triad and Frequency Triad tones, which they reliably did elsewhere.

## Concluding remarks

Our findings show that tones that are relatively long in duration will perceptually dominate relatively shorter, but more frequent, tones. The findings do not support theories stating that frequency of occurrence and duration information are logged by a single mechanism [45–47]. The ratings for durationally-biased tones changed differentially to the ratings for frequency-biased tones suggesting that more than one mechanism is needed. We did not test the possibility that there is a separate mechanism for every event that could accumulate in order.

Based on the results of previous research as well as of our experiments, we posit the following description of the perception of pitch structure. The organization of perceptual information will proceed according to whatever information is relevant, available, and most easily acquired: If the surface structure is recognized as being the same or similar to an existing schema, the schema will be used to organize perceptual structure [70, 113]. If surface structure does not match an existing schema, as in our experiments, information must be taken directly from the distributional properties of the sequences with relative duration being more salient, perhaps more easily perceived, and therefore treated as more important to perceived structure than frequency of occurrence or total duration. Relative duration is a simple comparison between the durations of different events, onset to offset, whereas frequency of occurrence (and total duration) require an accumulation across occurrences of events. Ease of acquisition would be important given a limited processing capacity perceptual system like that proposed by Kahneman [114].
